# Fine and Gray competing risk regression model to study the cause-specific under-five child mortality in Bangladesh

**DOI:** 10.1186/s12914-017-0112-8

**Published:** 2017-01-28

**Authors:** Khandoker Akib Mohammad, Most. Fatima-Tuz-Zahura, Wasimul Bari

**Affiliations:** Department of Statistics, Biostatistics & Informatics, University of Dhaka, Dhaka, 1000 Bangladesh

**Keywords:** Competing risk, Cumulative incidence function, Hazard function, Under-five mortality, Sub-distribution hazard ratio

## Abstract

**Background:**

The cause-specific under-five mortality of Bangladesh has been studied by fitting cumulative incidence function (CIF) based Fine and Gray competing risk regression model (1999). For the purpose of analysis, Bangladesh Demographic and Health Survey (BDHS), 2011 data set was used.

**Methods:**

Three types of mode of mortality for the under-five children are considered. These are *disease*, *non-disease* and *other causes*. Product-Limit survival probabilities for the under-five child mortality with log-rank test were used to select a set of covariates for the regression model. The covariates found to have significant association in bivariate analysis were only considered in the regression analysis.

**Results:**

Potential determinants of under-five child mortality due to disease is size of child at birth, while gender of child, NGO (non-government organization) membership of mother, mother’s education level, and size of child at birth are due to non-disease and age of mother at birth, NGO membership of mother, and mother’s education level are for the mortality due to other causes.

**Conclusion:**

Female participation in the education programs needs to be increased because of the improvement of child health and government should arrange family and social awareness programs as well as health related programs for women so that they are aware of their child health.

## Background

In developing countries, the study of under-five mortality is an important issue in public health programs. A country's level of socioeconomic development and quality of life are reflected by its under-five mortality rates. To monitor and evaluate population and health programs and policies, under-five mortality rates are used. The rates are also useful in identifying promising directions for health and nutrition programs in a country. Moreover, the Under-Five Mortality Rate (U5MR) for Bangladesh in 1989–93 was 133 per 1000 live births, while in 2007–2011 it decreased to 53 per 1000 live births [1–3]. It indicates that at the present mortality level, one in every 19 children dies before reaching his or her fifth birthday. However, in Bangladesh under-five mortality has long been very high as compared to the other countries in this South Asia. Though Bangladesh has made significant progress in reducing under-five mortality in recent years, it is still far below the related Millennium Development Goal (MDG) target, which is 46 per thousand live births.

Knowledge of causes of death among children under five is important because it helps to assess programmatic needs, prioritize interventions, and monitor progress. However, data on causes of death tend to be limited in Bangladesh. The vital registration systems are deficient in covering deaths occurring outside the health system, where cause of death is not reported. Verbal autopsy (VA) method is used for assessing the cause of death based on an interview with the next to kin or other caregivers who are knowledgeable about the events leading up to the death or who were present at the time of death [4]. A number of studies are found in which VA was used to determine the important causes of the child mortality in Bangladesh [5–9]. It is important to identify factors affecting the causes of child mortality and these factors have not been pointed out yet for Bangladesh. In this paper, an attempt has been made to identify the factors which may influence the causes of under-five mortality in Bangladesh.

Classical survival methods are not appropriate to analyze the time-to-event data in a complex situation like competing risk setup where an individual in the risk set is exposed to more than one causes for a failure [10–19]. One of the classical methods for analyzing the competing risk data is the proportional hazard (PH) model [20] to examine the effect of covariates on the cause specific hazard function. The major limitation of using PH model in a competing risk setup is that during estimation of regression parameters under a specific cause it considers the individuals failing from causes other than cause of interest as censored observations [21]. To overcome this limitation, Fine and Gray [22] developed a survival regression model based on cumulative incidence function (CIF) which describes the probability of occurring an event prior to a specific time. Unlike PH model, using CIF does not ignore the other competing risks when a specific cause is of interest [21, 23]. The proposed model is based on a proportional hazards model for the sub-distribution of a competing risk, where covariates directly affect the CIF. For right censored data, Fine and Gray [22] considered the inverse probability of censoring weighting (IPCW) technique for constructing the unbiased estimating equations for the regression parameters. Assuming stable covariate effects on the sub-distribution hazard rate over time, individual experiencing an event are left in the risk set forever but with decreasing weight to account for declining observability.

In this paper, CIF based survival regression model [22] has been employed to determine the potential risk factors for the causes of under-five child mortality using BDHS, 2011 data. This paper is organized as follows. Description of variables in BDHS, 2011 is given in Methods. In the same section, Fine-Gray competing risk regression model is briefly described. Results obtained from bivariate analysis as well as from the Fine-Gray regression models are shown in Results. This paper concludes in Discussion with a short discussion.

## Methods

To illustrate the Fine and Gray [22] model, in this paper, the under-five child mortality data extracted from BDHS, 2011 has been used. The data set is obtained from a two stage stratified sampling procedure, where in the first stage, 600 clusters were selected from both the rural (393 clusters) urban areas (207 clusters) of the country and in the second stage of sampling, a systematic sample of 30 households was selected on average from each enumeration area.

A total of 17,842 ever married women of age 12–49 from the selected households were interviewed to collect data on a complete history of their live births, including the sex, month and year of each birth, survival status and age at the time of the survey or age at death along with socio-economic and demographic variables. Moreover, information on deaths of children under age 5 in a household in the 5 years preceding the survey was collected from Verbal Autopsy Questionnaire (VAQ). A Verbal Autopsy Questionnaire (VAQ) was administered by the data collection team supervisor within a day of identification of the death if a child of under age 5 had died in a household in the 5 years preceding the survey. Verbal Autopsy dataset contains 490 observations with 12 variables.

The main purpose of this study is to determine the potential risk factors for the causes of under-five child mortality using a competing risk survival regression model. Children who were born preceding 5 years of the survey are considered for the study and hence a total of 8746 children are selected. Among these children, 408 died before reaching their fifth birthday. It indicates that under-five mortality rate is 46.65 per thousand children. That is, about one in every 21 children born in Bangladesh dies before reaching the fifth birthday. This data set underestimates the under-five mortality rate since 82 children were excluded from the data set as though they died under age of 5 years, they were born before 2006. The VA questionnaires used in BDHS, 2011 includes questions on the caretaker (or respondent) for the deceased child, age and place of death of deceased child, narrative history of events leading to death, prenatal care, labor, delivery, and obstetrical complications, accidental death, delivery history, description of signs and symptom preceding death, treatment preceding death, any direct, underlying, or contributing causes of death obtained from the death certificate. The assignment of cause of death of a child in BDHS, 2011 was defined differently than that in BDHS, 2004. In BDHS, 2004 the causes of deaths were assigned in a hierarchical process using computer algorithm [24], whereas a number of specially trained physicians were hired for this purpose who used the 2010 version of the International Classification of Deaths (ICD-10) for coding.

For the purpose of analysis, Bangladesh Demographic and Health Survey (BDHS), 2011 data set has been used, where causes of under-five child mortality were categorized as

**Table:** 

Disease	Neonatal tetanus, measles, diarrhea, pneumonia, meningitis, neonatal Jaundice, respiratory distress and serious infection
Non-disease	Congenital abnormality, drowning, birth asphyxia, birth injury, premature birth and malnutrition
Other causes	Other, undetermined and unspecific

On the basis of literature review on child mortality [5, 25–29] the covariates considered in this paper are: age of mother's at birth in years (<20, 20–30, >30), region (Barisal, Chittagong, Dhaka, Khulna, Rangpur, Rajshahi, Sylhet), mother's education level (No, primary, secondary, higher), wealth index (poor, middle, rich), birth order number (first birth, other), gender of child (male, female), type of place of residence urban, rural), place of delivery (hospital, other), exposure to media (yes, no), NGO (non-government organization) membership of mothers (yes, no) and size of child at birth (small, average/large). In BDHS 2011, size at birth is used as a proxy measure for weight at birth of child. Note that information on NGO membership of mother and exposure to media are not directly collected in BDHS, 2011 survey. The covariate exposure to media is created using information regarding reading newspapers, magazine, listening to radio and watching television. This covariate is selected as it promotes issues of maternal and child health programs. Moreover, the covariate NGO membership represents the membership if a respondent belongs to any of the NGOs like Grameen Bank, BRAC, BRDB, ASHA, Proshika etc. NGOs provide access to credit; play role in social intermediation and empowerment of women and act as entrepreneurs in delivery of social services and in commercial activities for poor people.

For analyzing competing risks data, standard survival analysis method, namely Cox PH model, has been commonly used. There exist a number of limitations in using this model. As remedy, Fine and Gray [22] proposed CIF based PH model to analyze survival data arising from a competing risk setup. In the competing risks setup, under each cause for the occurrence of an event of interest, a hazard function in the presence of covariates is considered. The number of failures from the causes other than the cause of interest reduces the actual number of failures from the cause of interest. Hence, influence the estimate of the probability of failure from the cause of interest [11]. To take care of this, Fine and Gray [22] developed a survival regression model using the CIF and sub-distribution hazard functions. The parameters involved in the model are estimated by incorporating weights in the partial likelihood function [30]. Under this model, for a covariate ${\mathrm{x}}_{\mathrm{r}}$, the sub-distribution hazard ratio (SHR) for the cause $\mathrm{j}$ $\left(\mathrm{j}=1,\cdots, \mathrm{p}\right)$ is given by $\exp \left({\upbeta}_{\mathrm{jr}}\right)$ keeping all other covariates at a fixed level, where ${\upbeta}_{\mathrm{jr}}$ is the regression coefficient [22, 31, 32].

To determine survival and under-five death rates by different covariates, sampling weights have been used. But as this paper aims to determine the potential risk factors for the causes of under-five child mortality, not to find any national level estimates, the design weights have not been considered in the regression analysis [33]. For the purpose of computation, R-package has been used [34].

## Results

It is observed from the data that more than half of the mothers (52.6%) gave their birth when they were in age group 20–30, while 34.7% of mothers gave birth at their young age (age is less than 20 years) and 12.7% of mothers became a mother of a child when they were older than 30 years. The distribution of children among the regions Barisal, Chittagong, Dhaka, Khulna, Rajshahi, Rangpur, Sylhet was 11.2, 20.0, 16.5, 11.2, 12.4, 12.7, and 16.1%, respectively. Among all women, 19.3% have no education while 30.7% percent have completed primary and 42.2% have completed their secondary education. Moreover, there are a few numbers of women (7.9%) who have completed their higher education. Most of the children are from poor families (41.7%), 19.0% from middle class and 39.3% from rich families. Among the children considered in the sample, 35.7% of them are the first child of their parents. The data set reveals that sex ratio is 106:100 indicating that 51.4% of children are male and 48.6% are female. The distribution of respondents with respect to place of residence is 69.5% and 30.5% for the rural and urban areas, respectively. In Bangladesh, it is found that maximum of mothers (72.8%) have not got hospital or clinical facilities during the delivery of their child. A large number of mothers (63.9%) were found to be exposed to media and 34.7% of mothers belonged to the NGO. At the time of delivery, only 17.6% of children were born small in size. In this paper, the event of interest is the death of a child under age of 5 years. An observation of a child is considered to be censored if that child survives until his/her fifth birthday.

The weighted survival and death rates under selected covariates are given in Table 1. Death rates due to different causes under different covariates are also given in Table 1. Out of 8746 children, only 4.5% children experienced the events. Among the children who failed to reach their fifth birthday, 47.8% died due to disease, 26.8% due to non-disease, and 25.4% due to other causes.

**Table 1:** Weighted under-five survival and death rates (per 100) due to different causes^a^ under selected covariates

Covariates	Sample Size	Survival	Cause of death		
			Disease	Non-disease	Other
Overall	8746	95.5	47.8	26.8	25.4
Age of mother at birth					
Age < 20 Age 20–30 Age> 30	3035 4600 1111	94.3 96.4 95.5	42.8 52.7 56.8	28.6 25.0 22.7	28.6 22.3 20.4
Region					
Barisal Chittagong Dhaka Khulna Rajshahi Rangpur Sylhet	977 1749 1444 981 1081 1107 1407	94.5 96.5 95.4 96.4 94.4 96.3 93.8	44.4 42.9 48.9 58.3 44.6 45.9 52.5	33.3 34.2 24.4 25.0 25.0 29.7 21.3	22.2 22.9 26.7 16.7 30.3 24.3 26.2
Mother's education level					
No education Primary Secondary Higher	1686 2681 3687 692	94.6 95.2 95.9 97.1	51.8 45.8 44.0 66.0	11.1 27.1 37.0 27.6	37.1 27.1 19.0 6.4
Wealth index					
Poor Middle Rich	3644 1661 3441	94.8 95.8 96.3	50.0 60.0 41.0	23.0 20.0 35.1	27.0 20.0 23.9
Place of residence					
Urban Rural	2669 6077	95.7 95.5	40.0 51.1	33.0 24.4	27.0 24.5
Gender of child					
Male Female	4499 4247	95.0 96.1	46.0 50.2	28.0 25.0	26.0 24.8
Birth order number					
First birth Others	3118 5628	94.9 95.9	45.1 51.2	29.4 24.4	25.5 24.4
Place of delivery					
Hospital Others	2382 6364	95.0 95.7	42.0 51.1	36.0 23.2	22.0 25.7
NGO membership of mother					
Yes No	3033 5713	94.5 96.1	43.6 51.3	31.0 23.1	25.4 25.6
Exposure to media					
Yes No	5585 3161	95.8 95.1	45.2 51.0	33.3 18.4	21.5 30.6
Size of child at birth					
Average/large Small	7203 1543	95.8 94.0	47.6 48.3	26.2 28.3	26.2 23.4

^a^Causes given in introduction

For the selection of potential covariates for the Fine and Gray survival models, survival experiences in different categories of a specific covariate have been explained by product-limit approach [35]. The associated log-rank test is used to determine whether a specific covariate has a significant impact on the under-five child mortality.

The covariates that have significant impact on the under-five child mortality are age of mother at birth, region, mother's education level, wealth index, birth order number, gender of child, NGO membership of mother and size of child at birth. Note that the *p*-values for these covariates are less than or equal to 0.05. To fit the Fine and Gray [22] competing risk survival regression model for identifying the potential determinants of causes of under-five mortality, covariates that are significantly associated with the under-five child mortality are only considered.

### Fine and Gray competing risk regression model

The sub-distribution hazard ratio (SHR) and 95% confidence interval (CI) of SHR for selected covariates obtained from Fine and Gray [22] competing risk survival regression model for different causes of under-five child mortality are given in Table 2.

**Table 2:** Sub-distribution hazard ratio (SHR) and 95% CI for SHR obtained from the Fine-Gray models for under-five mortality due to different causes^a^

Causes of under-five mortality	Disease		Non-disease		Other causes	
Covariates	SHR	95% CI of SHR	SHR	95% CI of SHR	SHR	95% CI of SHR
Age of mother at birth						
Age < 20 Age 20–30 Age> 30	1.28	0.86–1.92	1.45	0.84–2.51	2.42***	1.46–3.99
	Ref		Ref		Ref	
	1.18	0.78–1.81	1.27	0.67–2.43	0.73	0.36–1.48
Region						
Barisal Chittagong Dhaka Khulna Rajshahi Rangpur Sylhet	Ref		Ref		Ref	
	0.64 0.93 0.90 0.97 0.77 1.33	0.36–1.13 0.54–1.59 0.50–1.63 0.56–1.69 0.43–1.38 0.80–2.22	0.85 0.73 0.61 0.91 0.69 0.91	0.45–1.63 0.36–1.47 0.27–1.34 0.45–1.84 0.33–1.43 0.46–1.81	0.72 0.90 0.43 1.06 0.68 1.27	0.34–1.52 0.43–1.89 0.15–1.23 0.50–2.24 0.30–1.52 0.62–2.59
Mother's education level						
No education Primary Secondary Higher	Ref		Ref		Ref	
	0.76 0.70 0.57	0.51–1.13 0.45–1.09 0.25–1.32	1.93 2.01* 0.88	0.98–3.77 1.01–4.00 0.26–3.02	0.54** 0.32*** 0.15**	0.33–0.88 0.17–0.58 0.03–0.68
Wealth index						
Poor Middle Rich	0.90 Ref	0.62–1.31	1.48 Ref	0.84–2.62	1.10 Ref	0.60–1.97
	0.67	0.44–1.02	1.56	0.88–2.76	1.35	0.74–2.46
Birth order number						
First birth Others	1.03 Ref	0.68–1.56	1.37 Ref	0.80–2.33	1.05 Ref	0.62–1.77
Gender of child						
Male Female	1.21 Ref	0.91–1.60	1.54* Ref	1.05–2.26	1.24 Ref	0.83–1.83
NGO membership of mother						
Yes No	1.16 Ref	0.86–1.55	1.83*** Ref	1.23–2.70	1.53* Ref	1.01–2.32
Size of child at birth						
Average/large Small	0.63*** Ref	0.45–0.86	0.56** Ref	0.36–0.85	0.79 Ref	0.49–1.26

Ref: reference category, ***p-value<0.001, **p-value<0.01, *p-value<0.05, ^a^causes given in introduction

It is clear from Table 2 that for the under-five child mortality due to other causes, the hazard rate of children whose mothers are aged below 20 years during the pregnancy termination is 2.42 times as likely as the hazard rate of children whose mothers are aged between 20 and 30 years. This influence is statistically significant as *p*-value is <0.001. Moreover, children from the mothers in age group above 30 are likely to have same failure experience as the children from mothers in age group 20–30, but this is not a significant factor for the child mortality.

It is found that region and birth order number of index child do not have any significant influence on the any causes of under-five child mortality. Children of mothers having completed secondary education are significantly at a higher risk of mortality with *p*-value less than 0.05 relative to the children of mother having no education under the cause non-disease. It reveals from Table 2 that under-five child mortality occurs more frequently (with *p*-values less than 0.01) for children whose mothers are uneducated compared to children from educated mothers for other causes. Male children are at more risk of mortality due to non-disease (*p*-value < 0.05) than female children. Same pattern has also been observed for the children whose mothers are the members of NGO (*p*-value <0.001). For disease and non-disease average/large child has significantly (*p*-value <0.001) 37% and 44.0% lower risk of failure than small child in size respectively.

To examine how covariates considered for under-five mortality are associated with the infant mortality due to different causes, in this paper, Fine and Gray survival regression model has also been employed. The results obtained for infant mortality were given in Appendix (Table 3). Like under-five mortality, size of child at birth was found to have significant influence on infant mortality due to disease. Results obtained from infant mortality were similar to that of under-five mortality. Covariates found to have significant association with under-five mortality due to other causes were also found significant for infant mortality.

## Discussion

The study of under-five child mortality has become one of the most important researches of the developing countries including Bangladesh because of high rate of under-five child mortality. However, Bangladesh has witnessed a large decline in under-five mortality during the last decade [3]. The reduction of under-five mortality indirectly helps in reducing fertility by decreasing the desired number of children to be born due to increased probability of survival of a child. The under-five mortality is a composite index reflecting social, economic, health care facilities and delivery situation on one hand and maternal as well as family and community customs and practices on the other.

In this paper, an attempt has been made to determine the potential determinants for the causes of under-five child mortality in Bangladesh by using cumulative incidence function (CIF) based Fine and Gray [22] competing risk regression model and for the purpose of analysis, BDHS, 2011 data set was used. The causes of under-five child mortality are classified into three broad classes, which are disease, non-disease, and other causes. To select the set of covariates for the regression model, first a bivariate survival analysis using product-limit approach has been considered to examine the link between under-five child mortality and the selected covariates such as age of mother at birth in years, region, mother's education level, wealth index, birth order number, gender of child, type of place of residence, place of delivery, exposure to media, NGO membership of mothers and size of child at birth. Moreover, the associated log-rank test confirms that age of mother at birth, region, mother's education level, wealth index, birth order number, gender of child, NGO membership of mother and size of child at birth provides significant influence on under-five child mortality and these covariates are only considered in the regression analysis.

For the disease as a cause of under-five child mortality, the size of child at birth has been found to be potential determinant. Similar result was also found from the non-competing risk model to analyze mortality data [28, 36]. Children whose size at birth is average or large in size are at less risk of dying from disease than children with small size at birth. The covariates that have significant influence on the under-five child mortality due to non-disease are gender of index child, NGO membership, mother’s education level, and size of child at birth. Under-five mortality is higher for boys than for girls because baby boys are more vulnerable than baby girls from the time of conception. It is also found that under-five mortality is higher for the children whose mothers have NGO membership. A mother involved in NGO is poor and involves in many income general activities. For this reason, she cannot take proper care of her child and she has to face many difficulties in the time of her pregnancy. At the time of her pregnancy, she has to do many difficult works and maternity leave is not available in many cases. Surprisingly, mothers having primary or secondary education found to experience higher under-five mortality compared to mothers with no education. This may happen because those mothers involve with works outside keeping their children under attendant at home. Age of mother at birth and education level of mother are found to be inversely related with the under-five child mortality due to other causes. It is observed that mortality rate is higher for the children whose mothers in young age group. This is because younger mothers are comparatively less careful during pregnancy period and also have little knowledge about the proper growth of children. As a result, children suffer from malnutrition which causes poor immunity power resulting a high risk of mortality. It is also found that higher level of education attainment is associated with lower mortality risks because education exposes mothers to get information about better pregnancy and child health care. Like mortality due to non-disease, children whose mothers are member of NGO are more prone to mortality before reaching their fifth birthday than children whose mothers are not member of NGO. In literature, mother’s education level, region, wealth index, gender of child and size of child at birth were also found as potential factors for under-five mortality in non-competing risk model [25, 26, 36].

On the basis results obtained from this study, following recommendations can be suggested to the policy makers and government to reduce the under-five child mortality in Bangladesh. Female participation in the education programs needs to be increased because it consequently brings improvement in child health as well as in her life. Government should introduce education programs that increase people’s awareness regarding maternal and health care during pregnancy and child health care practices. Government should also arrange family and social awareness programs as well as health related programs for women so that they are aware of their child health.

## Conclusion

Under-five mortality due to different causes has been analyzed under a competing risk setup, which is the main strength of this paper. A nationally representative data has been used for the purpose of analysis. Only children born preceding 5 years of survey have been considered in the analysis to minimize recall bias and to reflect the recent mortality pattern.

In this paper, the analysis has been done under the assumption that the failure times are independent. From the data it reveals that some children belong to the same mother and hence times obtained from those children are likely to be correlated. This correlation needs to be taken into account to get more precised estimate of the parameter of interest. Though BDHS 2014 dataset is available, this dataset has not been used for the analysis since VA data are not available in it.

## Appendix

**Table 3:** Sub-distribution hazard ratio (SHR) and 95% CI for SHR obtained from the Fine-Gray models for infant mortality due to different causes^a^

Causes of infant mortality	Disease		Non-disease		Other causes	
Covariates	SHR	95% CI of SHR	SHR	95% CI of SHR	SHR	95% CI of SHR
Age of mother at birth						
Age < 20 Age 20–30 Age> 30	1.35 Ref	0.89–2.03	1.33 Ref	0.72–2.43	2.24** Ref	1.32–3.79
	1.10	0.69–1.71	1.49	0.75–2.94	0.71	0.33–1.49
Region						
Barisal Chittagong Dhaka Khulna Rajshahi Rangpur Sylhet	Ref		Ref		Ref	
	0.70 1.09 1.10 1.09 0.87 1.60	0.38–1.28 1.21–3.76 0.59–2.04 0.58–2.02 0.47–1.62 0.93–2.74	0.79 0.73 0.66 0.89 0.73 0.98	0.38–1.61 0.33–1.57 0.28–1.52 0.40–1.94 0.32–1.62 0.46–2.04	0.72 0.86 0.46 1.14 0.75 1.13	0.32–1.56 0.39–1.86 0.16–1.33 0.52–2.46 0.32–1.70 0.53–2.41
Mother's education level						
No education Primary Secondary Higher	Ref		Ref		Ref	
	0.81 0.70 0.58	0.54–1.21 0.43–1.10 0.23–1.41	1.74 2.09* 1.00	0.83–3.62 0.99–4.37 0.28–3.52	0.48*** 0.29*** 0.14***	0.28–0.81 0.15–0.52 0.02–0.62
Wealth index						
Poor Middle Rich	1.00 Ref	0.68–1.47	1.48 Ref	0.77–2.80	0.95 Ref	0.52–1.73
	0.69	0.44–1.07	1.60	0.85–3.01	1.29	0.70–2.36
Birth order number						
First birth Others	1.03 Ref	0.67–1.58	1.57 Ref	0.87–2.79	1.21 Ref	0.69–2.08
Gender of child						
Male Female	1.28 Ref	0.95–1.71	1.68* Ref	1.09–2.57	1.22 Ref	0.80–1.83
NGO membership of mother						
Yes No	1.18 Ref	0.88–1.58	1.84** Ref	1.18–2.82	1.57* Ref	1.01–2.42
Size of child at birth						
Average/large Small	0.67* Ref	0.47–0.93	0.47*** Ref	0.29–0.73	0.74 Ref	0.45–1.21

Ref: reference category, ****p*-value<0.001, ***p*-value<0.01, **p*-value<0.05, ^a^causes given in introduction
